# Fighting Sharka in Peach: Current Limitations and Future Perspectives

**DOI:** 10.3389/fpls.2016.01290

**Published:** 2016-08-30

**Authors:** Marco Cirilli, Filippo Geuna, Anna R. Babini, Valentina Bozhkova, Luigi Catalano, Beniamino Cavagna, Sylvie Dallot, Véronique Decroocq, Luca Dondini, Stefano Foschi, Vincenza Ilardi, Alessandro Liverani, Bruno Mezzetti, Angelantonio Minafra, Marco Pancaldi, Tiziana Pandolfini, Thierry Pascal, Vito N. Savino, Ralph Scorza, Ignazio Verde, Daniele Bassi

**Affiliations:** ^1^Department of Agricultural and Environmental Sciences (DISAA), University of MilanMilan, Italy; ^2^Phytosanitary Service, Regione Emilia-RomagnaBologna, Italy; ^3^Department of Breeding, Genetic Resources and Biotechnology, Fruit-Growing InstitutePlovdiv, Bulgaria; ^4^Centro Interprofessionale per le Attività VivaisticheRoma, Italy; ^5^Phytosanitary Service, Regione LombardiaMilano, Italy; ^6^INRA, UMR 385 BGPIMontpellier, France; ^7^INRA, Université de Bordeaux, UMR 1332 Biologie du Fruit et PathologieVillenave d’Ornon, France; ^8^Dipartimento di Scienze Agrarie, University of BolognaBologna, Italy; ^9^Centro Ricerche Produzioni VegetaliCesena, Italy; ^10^Consiglio per la Ricerca in Agricoltura e l’Analisi dell’Economia Agraria, Centro di Ricerca per la Patologia VegetaleRome, Italy; ^11^Consiglio per la Ricerca in Agricoltura e l’Analisi dell’Economia Agraria, Unità di Ricerca per la Frutticoltura di ForlìForlì, Italy; ^12^Dipartimento di Scienze Agrarie, Alimentari e Ambientali, Università Politecnica delle MarcheAncona, Italy; ^13^Istituto per la Protezione Sostenibile delle Piante (CNR-IPSP)Bari, Italy; ^14^Centro Attività VivaisticheFaenza, Italy; ^15^Department of Biotechnology, University of VeronaVerona, Italy; ^16^INRA, UR1052 GAFLMontfavet Cedex, France; ^17^Dipartimento di Scienze del Suolo, della Pianta e degli Alimenti (DiSSPA), Università degli Studi di Bari Aldo MoroBari, Italy; ^18^Appalachian Fruit Research Station, United States Department of Agriculture-Agricultural Research Service, KearneysvilleWV, USA; ^19^Consiglio per la Ricerca in Agricoltura e l’Analisi dell’Economia Agraria, Centro di Ricerca per la FrutticolturaRome, Italy

**Keywords:** genetic engineering, fruit breeding, PPV virus, *Prunus persica* (L.) Batsch, RNAi

## Abstract

Sharka, caused by *Plum Pox Virus* (PPV), is by far the most important infectious disease of peach [*P. persica* (L.) Batsch] and other *Prunus* species. The progressive spread of the virus in many important growing areas throughout Europe poses serious issues to the economic sustainability of stone fruit crops, peach in particular. The adoption of internationally agreed-upon rules for diagnostic tests, strain-specific monitoring schemes and spatial–temporal modeling of virus spread, are all essential for a more effective sharka containment. The EU regulations on nursery activity should be modified based on the zone delimitation of PPV presence, limiting open-field production of propagation materials only to virus-free areas. Increasing the efficiency of preventive measures should be augmented by the short-term development of resistant cultivars. Putative sources of resistance/tolerance have been recently identified in peach germplasm, although the majority of novel resistant sources to PPV-M have been found in almond. However, the complexity of introgression from related-species imposes the search for alternative strategies. The use of genetic engineering, particularly RNA interference (RNAi)-based approaches, appears as one of the most promising perspectives to introduce a durable resistance to PPV in peach germplasm, notwithstanding the well-known difficulties of *in vitro* plant regeneration in this species. In this regard, rootstock transformation to induce RNAi-mediated systemic resistance would avoid the transformation of numerous commercial cultivars, and may alleviate consumer resistance to the use of GM plants.

## Introduction

Sharka, caused by *Plum Pox Virus* (PPV), is the most devastating viral disease of peach and other stone fruits, resulting in significant economic losses (Cambra et al., 2006; Sochor et al., 2012). The progressive worldwide spread of this destructive disease requires a coordinated, focused effort to implement effective approaches to control its diffusion. Decades of research experience have made clear the need for transnational coordination involving not only researchers but all who are associated with the fruit industry including the phytosanitary services other than the growers themselves. An International Workshop on sharka disease in peach (Milan and Cesena, 2016, Italy^1^), gave the opportunity to review and update the state-of-art with particular attention to the main limitations and short-term perspectives.

The aim of this article is to provide a comprehensive picture of the current available possibilities to contrast sharka disease and to implement the resistance in peach, focusing on crucial aspects: the virus spread and management strategies within the EU regulatory framework; old and novel sources of genetic resistance (including the peach-related species almond and *Prunus davidiana*) and breeding perspectives; the application of novel genomics tools for the introgression of resistance and novel approaches for developing non-host resistance; the opportunity of using genetic engineering (GE) techniques, as already experienced in other fruit-tree species.

## Spread of Sharka Disease

Currently, PPV is spreading in many countries, with the occurrence of several strains with different epidemiology and specific infective capabilities. However, precise information and tracking of PPV distribution, correlated with the specific strain(s), is lacking in many peach growing regions. Little is known about the epidemiology of the Eastern Europe strains. Five new strains have been discovered in the last 10 years. The general framework of the PPV strains currently established includes, in addition to the most common PPV-M, -D and -REC, the strains -T (Turkey, Albania), -An (Albania), -EA (Egypt), -W (Canada, Ukraine, Russia, Latvia), -CR (Russia) and -C (Moldavia, Belarus, and Russia), the last two generally limited to cherry. Homologous recombination plays an important role in PPV evolution (Garcia et al., 2014). Several features including higher aphid transmission rates, a reduced latency period, faster virus diffusion in the orchards from primary infected plants and broad host range, confer to PPV-M the highest epidemicity in peach (Dallot et al., 2003). Information about the dynamics of host infection as it affects disease spread remains scarce.

Several strategies have been deployed to manage sharka disease, depending on the epidemic context. Eradication, based on orchard surveillance and removal of infected plants has been widely adopted in Western Europe and North America, whereas tolerant cultivars have been used in Eastern Europe (particularly for plum). In the former case, it becomes imperative to organize and carry out efficient strain-specific monitoring schemes implementing spatial–temporal modeling of disease spread. In France, surveillance intensity varies according to disease risk and control activities of the Plant Health Services are supported by skilled private professional organizations. The heterogeneity of PPV diagnostic tests is one of the main limitations for disease management. The test method(s) should be established by specific internationally agreed-upon rules. More rapid and accurate techniques for PPV diagnosis on candidate plants entering into the certification system are required, along with the ability to detect virus infection during the latency period. Novel techniques, such as Digital Droplet PCR (ddPCR; Gutiérrez-Aguirre et al., 2015) with the potential to detect up to one copy of viral RNA and isothermal amplification by reverse transcription-recombinase polymerase (Zhang et al., 2014), applicable directly on-field, are quite promising. To develop the next generation of novel diagnostics, close cooperation among all the interested actors, including entomologists will be important. For more information on PPV management strategies, see Rimbaud et al. (2015).

## Regulatory Framework

Considering the spread of sharka in Europe, the disease can no longer be controlled through quarantine legislation only, but the latter must be combined with the regulation of the Plant Propagating Material Quality. Evidence of such changes in the EU policies are found within documents in preparation which are now discussed in the context of the Harmonization of the Certification Scheme for Fruit Plants, particularly the Annex to the Commission Implementing Directive amending Annex IV Part A Section II of Directive 2000/29/EC on protective measures against the introduction in the Community of organisms harmful to plants or plant products and against their spread within the Community. Concerning PPV, many of the directives contained in the documents are not compatible with the epidemiology of the disease and its danger. For example, the time intervals provided for diagnostic tests are too long for an effective management. Another controversial and unexplained aspect is the criterion allowing the possibility to maintain mother plants under field condition in endemic areas.

The control of nursery plant material and trading pathways is critical. Insufficient controls on hundreds of new peach varieties offered to nurseries and growers every year, and the sporadic presence of PPV infected material in nurseries, along with the trade and/or exchange of uncontrolled materials for grafting by fruit growers, set up ideal conditions for national and international virus spread. Moreover, the release of the European Plant Passport according to the EU regulations and upon Phytosanitary Service control does not guarantee preventative testing of trade materials. In this regard the inspection and control on breeding and nursery materials, the utilization of virus-free (VF) certified plants, the restriction of nursery activities to pest-free areas or under screenhouse, and the safeguarding of pest-free areas are all imperative. For the management of sharka disease it is essential that:

(i) The effective and adequate application of a mandatory certification system for propagation material be used for all EU members;
(ii) The zone delimitation of European fruit growing areas based on PPV presence should limit the nursery production in open field only in virus-free areas (alternatively, in secure screen houses);
(iii) *Plum Pox Virus* isolates detected in new foci be characterized molecularly and serologically for risk assessment evaluation;
(iv) *Plum Pox Virus* infection status of any new cultivar released and its behavior toward PPV infection be evaluated at least for the most common PPV strains (M, D and Rec).

In conclusion, EU legislation must consider all these aspects since PPV should be regarded as a quarantine pest and the level of alert be enforced and not reduced.

## Source of Resistance to Sharka and Breeding Perspectives

The results of two decades of research confirm the absence of immune or resistant cultivars in peach germplasm, although a general low susceptibility to PPV-D strain has been reported (Rubio et al., 2012). The introgression of resistance from peach-related species, such as *Prunus davidiana* (Carrière; Pascal et al., 1998) and *Prunus dulcis* (Webb; Pascal et al., 2002; Martínez-Gómez et al., 2004), has been unsuccessful so far, mainly due to the difficulty in combining PPV resistance with traits suitable for the peach marketing (Moing et al., 2003). Most of the limitations come from the low rates of resistant individuals produced by crossbreeding and by the many unfavorable traits expressed by F_1_ hybrids, requiring at least several rounds of backcrossing to recover ‘commercial’ fruit traits. The absence of molecular markers associated with resistance, which would facilitate the selection of resistant seedlings, adds to the complexity of breeding PPV resistant cultivars. The introduction of resistance from *P. davidiana* has been recently suspended by French breeding programs in favor of the introgression from almond. As a result of the collaboration between INRA-Avignon (France) and CEBAS-CSIC (Spain) several almond cultivars resistant to PPV-M were identified, including ‘Del Cid,’ and have been chosen by INRA as resistant parents for building hybrid populations. Nevertheless, the experience within the Italian PPVCON project suggests that the use of almond as a resistance donor could suffer from the same limitations observed in resistant peach selections coming from UCD hybrids [(‘Padre’(almond) × ‘54P455’ (peach)) × ‘Hesse’ (peach) × self], characterized by poor fruit quality (Liverani et al., 2011).

Among other approaches attempted to confer sharka resistance, the use of aphid-resistant peach selections proved to be ineffective, since the trait was unable to ensure a protection against virus transmission in endemic areas (Liverani et al., 2015).

The extensive evaluation of more than 300 peach cultivars from core collections and recently released varieties showed the presence of a small number of accessions highly tolerant to the PPV-M strain (e.g., infectable by the virus but asymptomatic or developing only mild symptoms, particularly on fruit; Liverani et al., 2011). A promising resistant selection (e.g., ‘Spasena’) derived from the resistant parent ‘Dupnsika’ (Gabova, 1994) is currently under evaluation at the Fruit Growing Institute of Plovdiv (Bulgaria). Notably, the selection process occurring in open-field conditions within endemic sites, allows an increased reliability of resistance evaluation results. Indeed, as observed from field trials in Italian endemic areas, PPV-M was able to infect in just a few years about 70% of advanced selections, found resistant after several years of screenhouse ‘heavy test’ evaluations, i.e., grafting on already infected GF305 (Liverani et al., 2015). In perspective, the outdoor trials in endemic areas appear as a more practical, cost-effective, and reliable solution for the screening of promising selections.

An interesting research field is the use of resistant rootstocks, such as the almond cultivar ‘Garrigues,’ to induce resistance in the scion (Rubio et al., 2013). The postulated mechanism responsible for the prevention or recovery from infection is the systemic transmission by graft (almond) to scion (peach) of a silencing signal. Apart from the repeatability of the experiment in peach cultivars other than the already tested ‘GF305,’ some other questions are raised about its durability in time and in the field. Most important, considering the general tolerance of peach species to PPV-D strain, further studies should ascertain the stability of this mechanism against the most virulent PPV-M strain.

In conclusion, a divergent strategy for incoming breeding programs appears evident among some Italian and French research groups: the former is aiming at (short-term) development of cultivars highly tolerant to PPV-M (no symptoms, at least on fruit), suitable for the preservation of a peach industry in endemic areas; the latter attempts to introduce durable resistance (no virus replication in the tree) from almond (as PPV-M resistant parent or as a resistance-inducing rootstock). The introduction of tolerant plants is a matter of debate, since epidemiologists are concerned about the possible development of more aggressive PPV strains raising by recombination from mixed PPV infections and also for increasing the difficulties of implementing containment strategies on asymptomatic plants, since a PPV inoculum reservoir still persists in the area.

## Molecular and Functional Genomics

As demonstrated by several studies, the resistance to sharka conferred by the *P. davidiana* clone ‘P1908’ is of a quantitative nature, regulated by several QTLs with small effects, often variable across years (Decroocq et al., 2005). Linkage mapping experiments performed on progenies derived from different cross combinations of ‘P1908’ and/or SD hybrids (‘P1908’ × ‘Summergrand’) also demonstrated that QTL numbers and positions are affected by the genetic background of the peach parents (Marandel et al., 2009; Rubio et al., 2010). Currently, candidate gene(s) or molecular markers associated with PPV resistance have not been identified in *P. davidiana* or derived peach hybrids, hindering the short-term launching of marker-assisted selection (MAS) programs. The availability of the Peach Genome reference sequence and of a 9K SNP array platform (Verde et al., 2012), in addition to the availability of novel, powerful ‘omics’ tools may accelerate the identification of the genetic basis of PPV resistance in peach related species. For example, a Genotyping-By-Sequencing approach has been recently adopted to develop high-density genome markers for the introgression of resistance from almond (French FruitSELGEN project). While this approach is undoubtedly promising at the scientific level, concerns still persist about the real possibility of developing valuable cultivars from peach-almond hybrid(s) in the short-medium term.

The main bottlenecks for the identification of the genetic bases of PPV resistance, are still the cost, complexity and time-consuming phenotyping procedures, given the well-known unresolved issues:

(i) The lack of standardized phenotyping methods among research groups, i.e., ‘heavy test’ (see above) vs. ‘standard test’ (by inoculating buds from infected plants onto the accessions to be challenged), restricting the cross-validation of results.
(ii) A subjective and non-uniform interpretation of visual symptoms and discrepancies in terminology, particularly for ‘resistance’ and ‘tolerance.’ An alternative scoring system to overcome the strong subjectivity of symptoms evaluation is still lacking. A proposal for a standardized terminology is shown in **Table 1**.

**Table 1:** Proposal for a standardized terminology.

Classification	Visual sympthoms	ELISA	RT-PCR
**Non-host**			
Immune	-	-	-
**Host**			
Resistant	Absent	-	-
Susceptible	Absent (tolerant) to severe (sensitive)	±	+
Recovery	Absent	-	+ to -

*Immunity: non-host resistance (plant cannot be infected); Resistant: negative ELISA and RT-PCR assay, no visible symptoms; Susceptible: positive or negative ELISA, positive RT-PCR assay, symptoms on plant organs from absence (tolerant) to severe (sensitive) (mandatory specification of organ/tissue). ‘Recovery reaction,’ characterized by an initial low infection (RT-PCR positive only) later followed by no detectable infection (RT-PCR negative).*

(iii) The long-term requirement of evaluation trials for a reliable assessment of resistance. No quick protocol for resistance evaluation has been implemented so far and is not recommended, since the alternance of resting and growing periods is obviously important for the reliability of the trial.
(iv) The reliability of ‘in-field’ resistance evaluation vs. ‘screenhouse’ testing.

Knowledge of the mechanism of PPV-peach interaction at physiological, proteomic, metabolic and gene expression level are still scarce, although it may have practical implication for disease management (reviewed by Clemente-Moreno et al., 2015) or for the development of biomarkers to detect early PPV infection, especially for nursery mother trees. Recently, proteome analysis of PPV-infected peach plant showed that infection affected the abundance of proteins related to photosynthesis, carbohydrate, and amino acid metabolism (Clemente-Moreno et al., 2013). RNA-seq of peach leaf transcriptome after PPV-D infection demonstrated the complexity of plant response and the critical role of early responsive genes as a reaction against virus replication and translocation, before symptoms development (Rubio et al., 2015). Unfortunately, the experiments above were not performed on PPV resistant accessions, thus there are not information about candidate genes involved in sharka resistance in peach so far.

Developing non-host resistance in peach is a new frontier of research. Viruses encode a limited number of essential proteins, since recruit plant proteins (host factors) for every stage of the infection cycle (van Schie and Takken, 2014; García and Pallás, 2015). Gene(s) coding for such factors can be considered as susceptibility (S) genes and their mutation or loss of function limit the ability of the pathogen to cause disease. For example, recessive resistance to some *Potyviruses*, PPV included, is associated with mutations of genes belonging to the eukaryotic translation initiation factors (*eIF*) family in several plant species, thus representing an excellent candidate to also confer non-host resistance in peach. Different tools have been deployed to explore the possibility of developing a non-host resistance in peach:

(i) EcoTILLING approach, searching for peach variants in which susceptibility gene(s) are deleted or non-functional. Nevertheless, as a consequence of reduced genetic diversity, peach shows the lowest number of variants in comparison with other *Prunus* species. All promising individuals are hybrids (peach crossed to almond or peach wild related species);
(ii) TILLING approach, by creating *de novo* non-functional host genes through chemical mutagenesis (EMS). Three thousand individuals derived from the susceptible peach rootstock ‘GF305’ are currently under screening by whole-genome re-sequencing to evaluate the mutation rate after EMS treatment and the mutation rate in susceptibility genes.
(iii) S gene(s) silencing and/or Genome Editing (discussed in the next section).

The application of novel ‘omics’ approaches holds great promise for unraveling the complexity of the genetic mechanisms regulating sharka resistance. This knowledge is essential for developing useful molecular tools in breeding PPV resistant varieties as well as for implementing more efficient disease management strategies. The development of non-host resistance in peach is an ambitious target, that is now moving its first steps.

## Introducing Resistance in Peach By Genetic Engineering

In light of the complexity of breeding PPV-resistant peach cultivars by conventional strategies, as determined by the absence of resistant cultivars, by the low genetic diversity in peach germplasm, and by the ineffectiveness of introgression from related species, genetic engineering (GE) approaches appear an alternative strategy at the current status of knowledge (Ilardi and Tavazza, 2015).

Long-term evaluations, lasting over 20 years, in both screenhouse and endemic areas throughout Eastern Europe, have demonstrated the effectiveness of GE approaches to confer stable and durable sharka resistance in plum (Scorza et al., 2016). PPV resistance in transgenic ‘HoneySweet’ plum is based on RNA interference (RNAi), triggered by a complex multi copy insertion of an inverted repeat/hairpin configuration of the PPV coat protein (PPV-CP) transgene, activating the production of 25–26 nt class siRNA and ultimately leading to viral RNA degradation (Kundu et al., 2008). A more detailed knowledge of PTGS mechanisms has led to the development of more effective tools for RNAi-mediated engineered resistance. In particular, intron hairpin RNA (ihpRNA) constructs have proven to be highly effective inducers of local and systemic resistance against PPV in both model species *Nicotiana benthamiana* (Pandolfini et al., 2003; Di Nicola-Negri et al., 2005) and *Prunus domestica* (Hily et al., 2007; Monticelli et al., 2012). In addition, PPV-derived ihpRNA constructs were able to induce high resistance to a wide range of PPV strains (Di Nicola-Negri et al., 2010; Ravelonandro et al., 2014) also under variable abiotic and biotic conditions that are known to have a negative impact on gene silencing in plants (Di Nicola et al., 2014). Advantages of ihpRNA constructs include the high flexibility (it is possible to target multiple sequences from different PPV genomic regions or different PPV strains), high stability of siRNAs production, no documented interference with the endogenous RNA silencing machinery, robust and durable antiviral resistance and absence of transgene-derived proteins (Wang et al., 2013). Some questions related to undesirable consequences of RNAi still remain, such as off-target effects leading to changes in the host transcriptome; trade-off between defense and growth/development processes; and virus escape from silencing (Fuentes et al., 2016). However, the risk of such off-target effects can be identified by a well-developed science based risk assessment and reduced by a continuous monitoring as defined in a proper post-monitoring program to be applied after the release of the new events.

However, the application of genetic transformation techniques in peach has been limited by the difficulties in developing efficient regeneration and transformation protocols. Nevertheless, a protocol for *in vitro* regeneration via organogenesis, has been already developed for one of the most widely used peach rootstocks ‘GF677’ (Sabbadini et al., 2015). The protocol may be further improved by using different selection markers since ‘GF677’ appears to be naturally rather resistant to the antibiotic kanamycin. At present, the availability and the already demonstrated capabilities of many viral-derived constructs to induce RNAi against PPV, jointly with a high probability of transforming ‘GF677,’ make the hypothesis of rootstock engineering one of the most feasible and promising for peach cultivars (MIUR – PRIN VIRES project). Root-to-scion (and scion-to-root) siRNAs transfer has been already demonstrated in several annual model plants (reviewed in Pyott and Molnar, 2015). Rootstock transformation would be appealing, for several reasons: the scion would maintain its genetic background; no gene flow, because pollen and seeds would not be allowed to be produced by the genetically modified rootstock; the same transgenic rootstock can be used for many cultivars, avoiding the transformation of each single accession; and it may simplify many aspects related to the opinion of the consumers on GM plants (Lemgo et al., 2013). Recently, this approach has been successfully applied in a cherry rootstock to induce RNAi-mediated systemic resistance to Prunus Necrotic Ringspot Virus (Song et al., 2013; Zhao and Song, 2014), although the stability and durability of this approach requires further evaluation. On the contrary, transmission of RNA silencing was not observed in non-transgenic scions in apple (Flachowsky et al., 2012), and thus, additional research is required to set-up the appropriate strategies for an efficient silencing through grafting (Gohlke and Mosher, 2015).

While the ability of PPV-resistant ‘HoneySweet’ plum to transfer the silencing signal has not been demonstrated, the idea to use it directly as a rootstock in European peach growing areas is attractive. The main limitation arises from the implementation of experimental field trials, which require the approval of competent authorities. ‘HoneySweet’ and other reported GE and conventional PPV resistant *Prunus* should be field tested as rootstocks (and as scions) in PPV endemic areas. This work requires that competent authorities evaluate GE field tests on scientific merit and realistic biosafety issues, and that local authorities guarantee the safety of these plantings against destruction by radical GE opponents.

In spite of the success obtained through GE approaches to induce virus resistance in several crops, public concerns over the potential ecological impact of GE organisms and/or products strongly limit their use in Europe. In this sense, the approval of ‘HoneySweet’ by US authorities is a paradigm shift for fruit trees in terms of the application of GE technology (Scorza et al., 2013). Risk assessment is an integral part of GE plant production, and the time and costs that it requires should be added to those of plant transformation, selection and evaluation. In addition to the molecular analysis of plants, the biochemical characterization of fruits and the evaluation of resistance, further information may be required from authorities and consumers, including plant-virus and plant-insect interactions, gene flow and the potential risk of off-target gene silencing (all of which were addressed for ‘HoneySweet’ in the U.S. regulatory dossiers). Excluding marker proteins, the risks associated with newly expressed proteins (i.e., allergenicity or toxicity) would not be meaningful for RNAi-based engineering, due to the lack of viral proteins produced by RNAi plants.

Genome editing is a novel and promising type of GE, based on the use of engineered nucleases for the introduction of mutations at target genomic sequences. Despite the growing positive opinion about its application from authorities and consumers, it is still hardly applicable in peach. In order to maintain clonal stability in vegetative propagated plants, the application of genome editing techniques requires the development of effective protocols of plant regeneration from protoplast culture, unavailable in peach. Lacking the possibility of transient expression, stable transformation with the Cas9 system will be necessary (Xu, 2013; Jia and Wang, 2014), which will then require a round of backcrossing or self-pollination for its removal after editing procedures and regeneration. The mandatory germline transition generates a novel cultivar that must then be evaluated before market introduction. In addition, some concerns still persist about how this procedure of developing non-transgenic plants using GE techniques would be classified by the authorities. Despite this, the resistance conferred by the editing of susceptibility genes (S) to confer PPV resistance is an interesting perspective. The *eIF4E*(s), *cPGK, DBP1* genes, and/or *PpDDXL* recently functionally characterized in peach represent good candidates for the application of Genome Editing (Huang et al., 2010; Castelló et al., 2011; Poque et al., 2015). However, because S genes have a function other than as a compatibility factor for the pathogen, the side effects caused by their mutation demand a one-by-one assessment of their usefulness for application. Plant regeneration from protoplast cell lines has never been demonstrated in peach and this remains the major issue for the application of genome editing in heterozygous vegetatively propagated plants. Therefore, current knowledge of RNAi approaches seem more suitable to yield positive results.

A relatively recent technology for rapid cycle breeding (‘FastTrack’) developed in other species (apple, plum, etc.) is a breeding system that uses a GE tree expressing a flowering pathway gene, such as *FT* gene orthologs, to obtain fruiting trees in 1 year (or even less) (Flachowsky et al., 2011; Srinivasan et al., 2012). Shortening the juvenile stage in peach would accelerate the conventional breeding procedures for the introgression of sharka resistance from peach-related species. Also, the final resulting trees would not be GE unless the PPV resistance gene used is a transgene.

## Conclusion

The containment of sharka disease spread is one of the most important priorities of the European peach industry. The complexity of this phytosanitary issue does not allow simple, rapid solutions. The application of preventive measure with the maximum alert level and, possibly, their reinforcement through the implementation of more effective management strategies, are of utmost importance. The adoption of internationally agreed-upon rules for diagnostic tests, strain-specific monitoring schemes and spatial–temporal modeling of virus spread, are all essential for a more effective sharka containment. The EU regulations on nursery activity should be modified based on the zone delimitation of PPV presence, limiting open-field production of propagation materials only to virus-free areas. Prevention should be combined with the introduction of genetic resistance in a reasonable time, but not at the expense of high fruit quality productions. A part from still to be verified existence of intraspecific sources of PPV resistance in peach, the complexity of introgression from related-species imposes the search for alternative strategies. Currently, the use of GE, particularly RNAi-based approaches, appears as one of the most promising perspectives, notwithstanding the well-known difficulties of *in vitro* plant regeneration in this species. Rootstock transformation to induce RNAi-mediated systemic resistance to PPV would avoid the transformation of numerous commercial cultivars, and may alleviate consumer concerns to the use of GM plants. In this regard, the use of GE approaches represent a fundamental opportunity, and as such it should be supported not only from a technical-scientific view point, but also in a broader socio-political context.

## Author Contributions

MC: Drafted the manuscript and critically revised it. FG and RS: Critically revised the manuscript. AB, VB, LC, BC, SD, LD, VD, SF, VI, AL, BM, AM, MP, TP, ThP, VS, and IV: Critically revised the manuscript for important contents. DB: conceived the manuscript and critically revised it. All authors read and approved the final manuscript.

## Conflict of Interest Statement

The authors declare that the research was conducted in the absence of any commercial or financial relationships that could be construed as a potential conflict of interest.

## Footnotes

1 https://sites.google.com/site/ppvsymposium2016
